# Therapeutic Effect of Iron Citrate in Blocking Calcium Deposition in High Pi-Calcified VSMC: Role of Autophagy and Apoptosis

**DOI:** 10.3390/ijms20235925

**Published:** 2019-11-25

**Authors:** Paola Ciceri, Monica Falleni, Delfina Tosi, Carla Martinelli, Stefania Cannizzo, Giulia Marchetti, Antonella D’Arminio Monforte, Gaetano Bulfamante, Geoffrey A Block, Piergiorgio Messa, Mario Cozzolino

**Affiliations:** 1 Renal Research Laboratory, Department of Nephrology, Dialysis and Renal Transplant, Fondazione IRCCS Ca’ Granda, Ospedale Maggiore Policlinico and Fondazione D’Amico per la Ricerca sulle Malattie Renali, 20100 Milan, Italy; p.ciceri@hotmail.it (P.C.); piergiorgio.messa@unimi.it (P.M.); 2 Department of Health Sciences, Division of Pathology, University of Milan, ASST Santi Paolo e Carlo, 20142 Milan, Italy; monica.falleni@unimi.it (M.F.); delfina.tosi@unimi.it (D.T.); carla.martinelli@unimi.it (C.M.); gaetano.bulfamante@unimi.it (G.B.); 3 Department of Health Sciences, Clinic of Infectious Diseases, University of Milan, ASST Santi Paolo e Carlo, 20142 Milan, Italy; stefania.cannizzo@unimi.it (S.C.); giulia.marchetti@unimi.it (G.M.); antonella.darminio@unimi.it (A.D.M.); 4 Nephrology, Denver Nephrolgysts PC, Denver, CO 80230, USA; geoff.block@reatapharma.com; 5 Department of Health Sciences, Renal Division, University of Milan, ASST Santi Paolo e Carlo, 20142 Milan, Italy

**Keywords:** iron, vascular calcification, apoptosis, VSMC, phosphate, autophagy

## Abstract

In chronic kidney disease (CKD), the first cause of mortality is cardiovascular disease induced mainly by vascular calcification (VC). Recently, iron-based phosphate binders have been proposed in advanced CKD to treat hyperphosphatemia. We studied the effect of iron citrate (iron) on the progression of calcification in high-phosphate (Pi) calcified VSMC. Iron arrested further calcification when added on days 7–15 in the presence of high Pi (1.30 ± 0.03 vs 0.61 ± 0.02; OD/mg protein; day 15; Pi vs Pi + Fe, *p* < 0.01). We next investigated apoptosis and autophagy. Adding iron to high-Pi-treated VSMC, on days 7–11, decreased apoptotic cell number (17.3 ± 2.6 vs 11.6 ± 1.6; Annexin V; % positive cells; day 11; Pi vs Pi + Fe; *p* < 0.05). The result was confirmed thorough analysis of apoptotic nuclei both in VSMCs and aortic rings treated on days 7–15 (3.8 ± 0.2 vs 2.3 ± 0.3 and 4.0 ± 0.3 vs 2.2 ± 0.2; apoptotic nuclei; arbitrary score; day 15; Pi vs Pi + Fe; VSMCs and aortic rings; *p* < 0.05). Studying the prosurvival axis GAS6/AXL, we found that iron treatment on days 9–14 counteracted protein high-Pi-stimulated down-regulation and induced its de novo synthesis. Moreover, iron added on days 9–15 potentiated autophagy, as detected by an increased number of autophagosomes with damaged mitochondria and an increase in autophagic flux. Highlighting the effect of iron on apoptosis, we demonstrated its action in blocking the H_2_O_2_-induced increase in calcification added both before high Pi treatment and when the calcification was already exacerbated. In conclusion, we demonstrate that iron arrests further high Pi-induced calcium deposition through an anti-apoptotic action and the induction of autophagy on established calcified VSMC.

## 1. Introduction

Vascular calcification (VC) is common in advanced chronic kidney disease (CKD), and is associated with poor cardiovascular (CV) outcomes [1]. The pathogenesis of VC is multifactorial and incompletely understood. CKD patients are at risk of VC because of multiple risk factors. High phosphate (Pi) levels are associated with a high risk of VC and CV disease in the general population and, in particular, in CKD patients [2]. It has been clearly demonstrated that high Pi induces vascular smooth muscle cells (VSMCs) osteoblastic differentiation [3]. Beside high Pi, other factors potentially cause the phenotypical change of VSMCs into an osteoblast-like cell, such as high total body burden of calcium (Ca) and Pi, low levels of circulating and locally-produced inhibitors, impaired renal excretion, and CKD-MBD (Mineral Bone Disorder) treatments [4].

Commonly, treatment of hyperphosphatemia requires a multimodal approach: Dialysis, dietary restriction of Pi intake (i.e., food with low Pi content, balancing sufficient protein intake and restricting Pi intake, counteracting “hidden” phosphate due to insufficient labelling of processed food), and the prescription of Pi binders [5]. Considerations for the choice of Pi binder may include age, gender, diabetes, low bone turnover, vascular and/or valvular calcification, and inflammation [6]. Recently, two new iron-based Pi binders have become available to dialysis patients to treat hyperphosphatemia [7]. In addition, we demonstrated: (1) the effect of iron citrate in preventing calcification in an in vitro model of high Pi-induced VC, finding that iron reduces Ca deposition through the prevention of apoptosis and the enhancement of autophagy [8]; (2) that iron is able to both prevent and partially revert extracellular matrix osteo-chondrogenic shifts induced by high Pi treatment acting on elastinolysis, fibrosis, mucins synthesis, and glycolytic VSMC metabolism [9]. Although the effect of iron citrate on the progression of calcification in established calcified VSMCs has already been presented in a previous study of our research group [8], the aim of the present set of data is to better elucidate the mechanisms of the direct effect of iron citrate on the progression of calcification in established high Pi-calcified VSMCs.

## 2. Results

### 2.1. Iron Citrate Arrests High Pi-Induced Calcium Deposition in Established Calcified VSMCs

**Figure 1:**
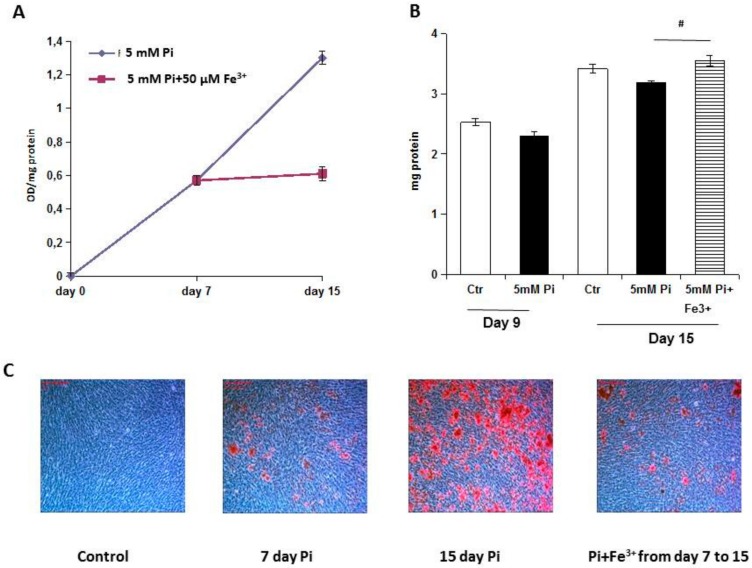
Effect of therapeutic addition of ferric citrate on high Pi-induced progression of calcification. Rat VSMCs were cultured with 5 mM Pi in a calcification medium for up to 15 days. (**A**) The addition of 50 µM Fe to the already calcified VSMCs from days 7 to day 15 was able to completely block additional high Pi calcium deposition (* *p* < 0.01). Calcium deposition was measured and normalized by cellular protein content. Data are presented as mean ±SE of five experiments in triplicate. (**B**) The addition of 50 µM Fe on already calcified VSMCs from days 7 to day 15 was able to improve VSMC viability, measured as the total protein content (# *p* < 0.05). Data are presented as the mean ±SE of five experiments in triplicate. (**C**) Ca deposition was visualized at a light microscopic level by Alizarin Red staining. Red indicates Ca deposits. The figure shows that treatment from days 7 to 15 of calcification with 50 µM Fe blocks calcium deposition that is comparable to seven. Magnification 200×. Representative results of one of the three different experiments.

Ferric citrate was used in an experimental setting of established calcified VSMCs to evaluate the effect of iron on the progression of calcification. In this setting, we added 50 µM ferric citrate (Fe) seven days after VSMCs had been challenged with 5 mM Pi (high Pi), and treated them with Fe and high Pi for the following eight days, i.e., up to day 15. As shown in Figure 1A, the therapeutic addition of Fe completely blocked the additional calcification induced by high Pi. In fact, from days 7 to 15, there was a significant progression of calcium deposition in high Pi-treated samples (0.57 ± 0.02 vs 1.30 ± 0.03, OD/mg protein; day 7 vs day 15, *p* < 0.01, Figure 1A), which were reduced by 95% by eight days’ treatment with 50 µM Fe (1.30 ± 0.03 vs 0.61 ± 0.02, OD/mg protein; day 15 Pi vs Pi + Fe days 7–15, *p* < 0.01, Figure 1A) to the same extent of calcification on day 7 (0.57 ± 0.02 vs 0.61 ± 0.02, OD/mg protein; day 7 Pi vs Pi + Fe days 7–15, ns, Figure 1A). Iron influenced not only calcium deposition but also VSMC viability, as shown by the protein content in samples treated with iron from days 7 to 15 compared to high Pi-challenged samples on day 15 (3.55 ± 0.09 vs 3.19 ± 0.03, mg protein; Pi + Fe days 7–15 vs day 7 Pi, *p* < 0.05, Figure 1B)

Evaluating the size and number of calcium granules, as expected, there were calcified deposits with different sizes after seven days of high Pi challenge that increased, becoming a confluent structure of deposits on the cellular layer on day 15 (0.8 ± 0.1 vs 2.7 ± 0.1; day 7 vs day 15; a.s.; *p* < 0.01; Figure 1C). Treatment with Fe from days 7 to 15 of calcification blocked the additional granule deposition with no detectable difference in the size or quantity of the granules compared with day 7 of calcification (day of start of iron treatment) (0.8 ± 0.1 vs 1.2 ± 0.2; Pi day 7 vs Pi + Fe days 7–15; a.s.; ns; Figure 1C).

### 2.2. Ferric Citrate Counteracts High Pi-Induced Apoptosis in Calcified VSMCs

The effect of ferric citrate on apoptosis was studied by the detection of the early marker phosphatidylserine via binding with annexin V, by nuclei fragmentation with TUNEL, by the effect on H_2_O_2_ exacerbation of high Pi-induced calcification and by the modulation of the prosurvival pathway GAS6/AXL.

**Figure 2:**
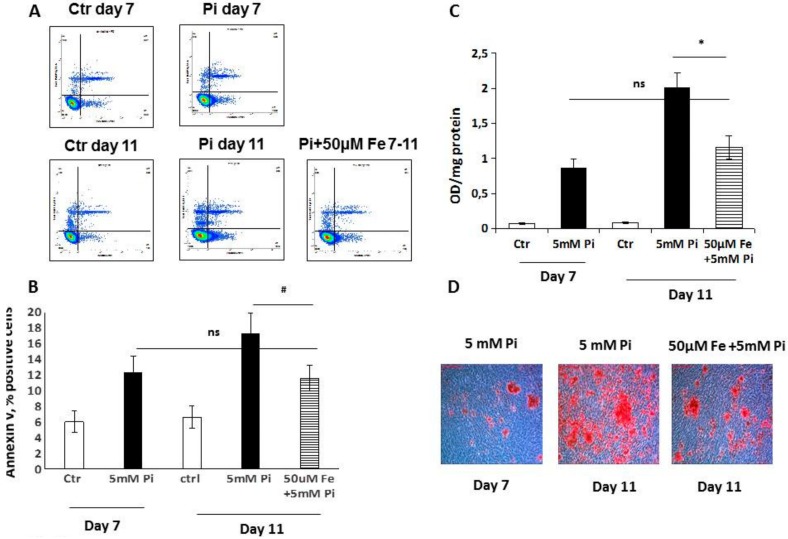
Effect of the therapeutic addition of ferric citrate on high Pi-induced progression of apoptosis. Rat VSMCs were cultured with 5 mM Pi in a calcification medium for up to 11 days. Apoptosis was evaluated by flow cytometry detection by annexin V and 7-amino-actinomycin D (7-AAD) staining. (**A**) Representative dot plots of 7-AAD versus annexin-V-PE. VSMC were left untreated (control) or were treated with Pi or Pi + Fe. The lower left quadrant contains the vital (double negative) population; the lower right contains the early apoptotic (annexin V+/7-AAD−) population; the upper right contains the late apoptotic/necrotic (annexin V+/7-AAD+) population; and the upper left contains the prenecrotic (annexin V−/7-AAD+) population. (**B**) Average percentage of early apoptotic and late apoptotic/necrotic cells. Treatment with 50 µM Fe from days 7 to 11 of calcification was able to significantly block the increase in high Pi-induced Annexin V positive cells (# *p* < 0.05, ns: No significance). Data are presented as mean ±SE of four different experiments. (**C**) The addition of 50 µM Fe on already calcified VSMCs from days 7 to day 11 was able to block further high Pi-induced calcium deposition (* *p* < 0.01). Calcium deposition was measured and normalized by cellular protein content. Data are presented as the mean ±SE of four experiments in triplicate. (**D**) Ca deposition was visualized at a light microscopic level by Alizarin Red staining. Red indicates high Pi-dependent Ca deposits. The images show that treatment from days 7 to 11 of calcification with 50 µM Fe blocked calcium deposition with results comparable to those of day 7. Magnification 200×. Representative result of one of four different experiments.

The treatment of calcified VSMCs from days 7 to 11 of calcification with iron was able to significantly decrease the percentage of annexin V positive cells, and thus apoptosis. Both 7 and 11 days of high Pi challenge induced a significant increase in apoptotic cell number compared to controls (6.0 ± 1.4 vs 12.3 ± 2.1 day 7, 6.6 ± 1.5 vs 17.3 ± 2.6 day 11, ctr vs Pi, annexin V% positive cells; *p* < 0.01, Figure 2A,B). Treatment for four days with 50 µM Fe, and from days 7 to 11 with calcification, was able to significantly block the increase in high Pi-induced apoptotic cell number, i.e., by 53% (17.3 ± 2.6 vs 11.6 ± 1.6; day 11; Pi vs Pi + Fe; annexin V% positive cells; *p* < 0.05; Figure 2A,B). In parallel, Fe treatment from days 7 to 11 of calcification significantly blocked additional high Pi-induced calcium deposition, i.e., similarly to the 7–15-day experimental protocol (74%; Figure 2C,D).

**Figure 3:**
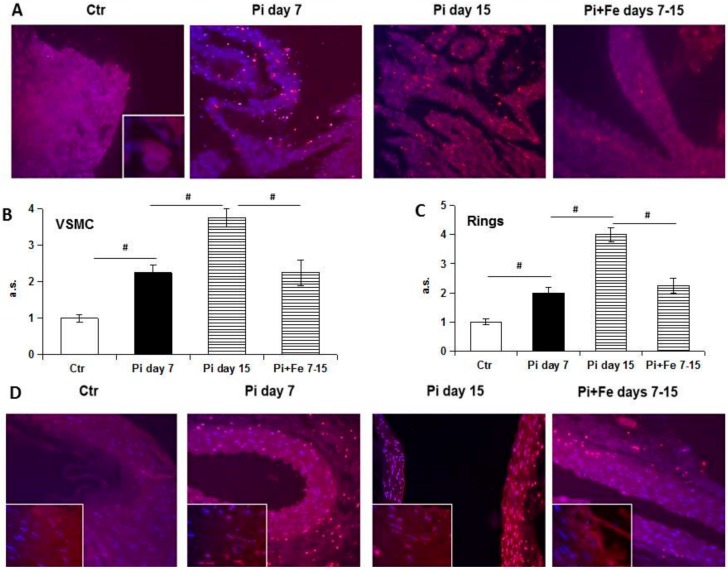
Effect of therapeutic addition of ferric citrate on 15 days of high Pi-induced progression of apoptosis in aortic rings and VSMCs. Rat VSMCs and aortic rings were cultured with high Pi (5 and 3 mM, respectively) in a calcification medium for up to day 15. (**A**,**D**) Apoptosis was visualized by TUNEL staining; (**B**,**C**): Semi-quantitative analysis of staining data. (**A**,**D**): Therapeutic addition of 50 µM Fe was able to block high Pi-induced nuclear apoptotic red signal progressive increase from day 7 to day 14, both in VSMCs and within the aortic wall (inset). (**B**,**C**) The addition of 50 µM Fe to already calcified VSMCs and aortic rings was able to protect the vessel from the high Pi-induced nuclei fragmentation detectable as red signals. Data are presented as the mean ±SE of at least two experiments in triplicate. (# *p* < 0.05). Magnification: Panel A 40×, panel D 20×, inset 100×.

We performed TUNEL staining to evaluate a late marker of apoptosis as nuclei fragmentation. From the images in Figure 3, it is possible to detect only sporadic apoptotic fragmented nuclei as a red signal in control VSMCs and aortic rings. Seven days of high Pi treatment induced apoptosis that progressed to diffuse fragmentation in almost all cell nuclei evaluated after 15 days of challenge, both in VSMCs and aortic rings (Figure 3A,B). Iron addition when calcification was established, after 7 days of high Pi treatment, induced a block in the progression of apoptosis. In fact, by analyzing on day 15 of high Pi challenge the apoptotic nuclei both in VSMCs and aortic rings treated with 50 µM Fe from day 7 to 15, there was significantly less apoptosis compared to that on day 15 (3.8 ± 0.2 vs 2.3 ± 0.3 and 4.0 ± 0.3 vs 2.3 ± 0.2; apoptotic nuclei; arbitrary score; day 15; Pi vs Pi + Fe; VSMCs and aortic rings, respectively; *p* < 0.05, Figure 3B,C), with no significant difference compared to day 7 (2.2 ± 0.2 vs 2.3 ± 0.3 and 2.0 ± 0.2 vs 2.3 ± 0.2; apoptotic nuclei; arbitrary score; Pi day 7 vs Pi + Fe days 7–15; VSMCs and aortic rings, respectively; ns Figure 3B,C).

**Figure 4:**
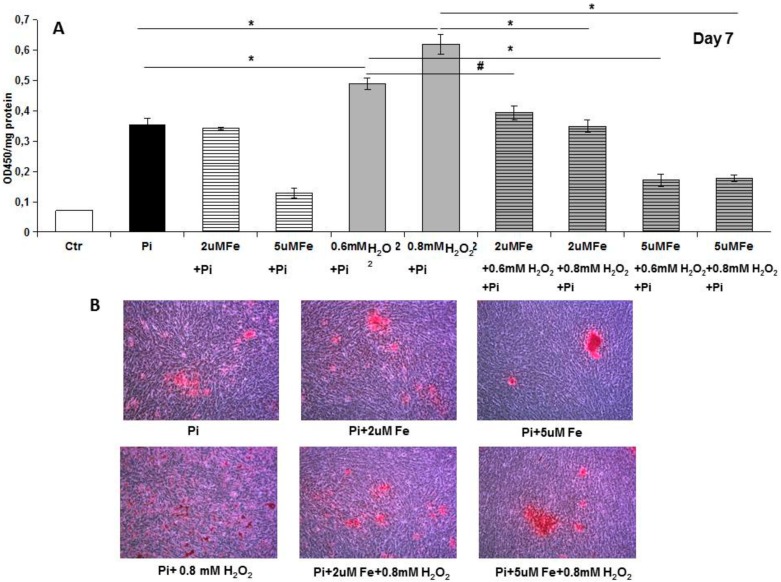
Effect of prophylactic addition of ferric citrate on H_2_O_2_ worsening of high Pi-induced calcification. Rat VSMCs were cultured with 4.5 mM Pi in the calcification medium for up to 7 days. (**A**) Pretreatment with H_2_O_2_ was able to worsen high Pi-induced calcium deposition. Iron addition (2 and 5 μM) completely prevents H_2_O_2_ worsening of high Pi calcification. Calcium deposition was measured and normalized by cellular protein content. Data are presented as the mean ±SE of three experiments in triplicate. (# *p* < 0.05; * *p* < 0.01). (**B**) Ca deposition was visualized at light microscopic level by Alizarin Red staining. Red color indicates Ca deposits. The figure shows images of calcium deposition modulation by iron in the presence of H_2_O_2_ in the experimental conditions indicated. Magnification 200×. Representative result of one of three different experiments.

**Figure 5:**
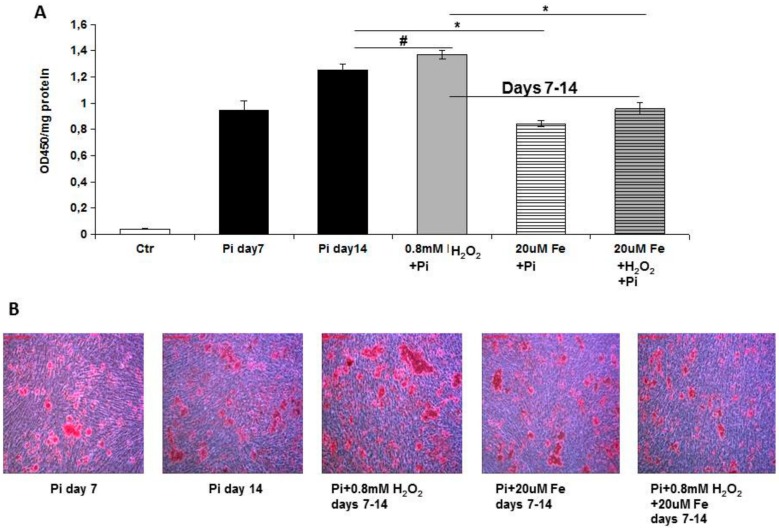
Effect of therapeutic addition of ferric citrate on H_2_O_2_ worsening of high Pi-induced calcification. Rat VSMCs were cultured with 4.5 mM Pi in a calcification medium for up to 14 days. (**A**) The addition of 20 µM Fe on already calcified VSMCs from day 7 to day 14 was able to completely block additional high Pi calcium deposition induced by proapoptotic molecule H_2_O_2_ (0.8 mM) treatment during the same period. Calcium deposition was measured and normalized by cellular protein content. Data are presented as the mean ±SE of three experiments in triplicate. (# *p* < 0.05; * *p* < 0.01). (**B**) Ca deposition was visualized at a light microscopic level by Alizarin Red staining. Red indicates Ca deposits. The figure shows images of calcium deposition modulation by iron in the presence of H_2_O_2_ in the experimental conditions indicated. Magnification 200×. Representative result of one of three different experiments.

In order to evaluate the effect of iron on apoptosis-induced calcification, we treated VSMCs with high Pi and a proapoptotic stimulus (H_2_O_2_) which was able to increase calcium deposition. Treatment with either 0.6 or 0.8 mM H_2_O_2_, starting on day 0 of calcification, resulted in +37% and +74% increases in calcium deposition, as expected (0.49 ± 0.02 and 0.62 ± 0.03 vs 0.36 ± 0.02; 0.6 and 0.8 mM H_2_O_2_ + Pi vs Pi, day 7; OD/mg protein; *p* < 0.01, Figure 4). Next, we added 2 and 5 μM iron, the first as a non-effective concentration and the second yielding a 60% inhibition of calcification in this experimental setting (Figure 4). Five micromolar Fe was able to completely abolish the H_2_O_2_-induced exacerbation of high Pi-driven calcium deposition (0.49 ± 0.02 vs 0.17 ± 0.02; 0.6 mM H_2_O_2_ + Pi vs 5 μM Fe + 0.6 mM H_2_O_2_ + Pi; and 0.62 ± 0.03 vs 0.18 ± 0.01; 0.8 mM H_2_O_2_ + Pi vs 5 μM Fe + 0.8 mM H_2_O_2_ + Pi; day 7; OD/mg protein; *p* < 0.01, Figure 4). Surprisingly, 2 μM Fe, that was ineffective on high Pi-induced calcium deposition, was also able to completely abolish the H_2_O_2_-induced worsening of calcification (0.49 ± 0.02 vs 0.39 ± 0.02; 0.6 mM H_2_O_2_ + Pi vs 2μM Fe + 0.6 mM H_2_O_2_ + Pi; and 0.62 ± 0.03 vs 0.35 ± 0.02; 0.8 mM H_2_O_2_ + Pi vs 2 μM Fe + 0.8 mM H_2_O_2_ + Pi; day 7; OD/mg protein; *p* < 0.01, Figure 4). We then chose a 0.8 mM H_2_O_2_ concentration to test whether iron was able to stop apoptosis-induced calcium deposition when calcification was already established. Adding H_2_O_2_ from day 7 to day 14 resulted in an exacerbation of calcification (1.25 ± 0.04 vs 1.37 ± 0.03; Pi vs 0.8 mM H_2_O_2_ + Pi day 7–14; day 14; OD/mg protein; *p* < 0.05, Figure 5). Treating VSMCs from day 7 to day 14 with 20μM Fe resulted in a block of not only high Pi increase in calcification (1.25 ± 0.04 vs 0.84 ± 0.03; Pi vs Pi + Fe day 7–14; day 14; OD/mg protein; *p* < 0.01, Figure 5), but also in an apoptosis-induced worsening of high Pi caused by H_2_O_2_ (1.37 ± 0.03 vs 0.96 ± 0.05; H_2_O_2_ + Pi day 7–14 vs Fe + H_2_O_2_ + Pi day 7–14; day 14; OD/mg protein; *p* < 0.01, Figure 5).

**Figure 6:**
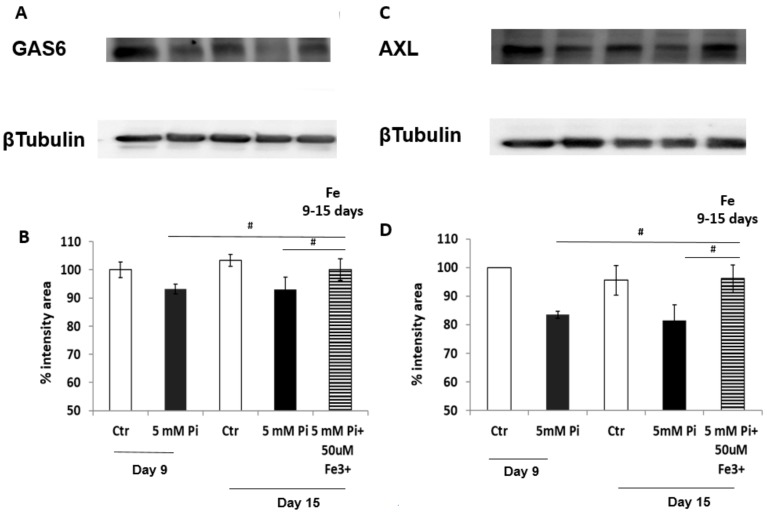
Effect of therapeutic addition of ferric citrate on the expression of the GAS6/AXL pathway during the progression of high Pi-induced calcification. Rat VSMCs were cultured with 5 mM Pi in a calcification medium for up to 15 days. **A** and **C**: GAS6 and AXL protein expression were analyzed by western blot. Lane 1: Control day 9; lane 2: 5 mM Pi day 9; Lane 3: Control day 15; lane 4: 5 mM Pi day 15; lane 5: 5 mM Pi + 50 µM Fe from day 9 to day 15. Representative result of one of three different experiments. **B** and **D**: Analysis of the intensity of the immunoblot bands of GAS6 and AXL normalized for beta tubulin bands are shown. Therapeutic addition of 50 µM Fe stimulates the synthesis of both GAS6 and the AXL protein, thereby inducing this prosurvival pathway. Data are presented as the mean ±SE of three different experiments (# *p* < 0.05).

We next evaluated the prosurvival pathway GAS6/AXL that, as expected, was down-regulated at both 9 and 15 days of calcification, with a significant decrease in protein expression (−8.45% and −8.55% GAS6; −14.29% and −16,27% AXL; Pi day 9 and 15 respectively, compared to control; *p* < 0.05; Figure 6). Treating calcified VSMCs from day 9 to day 15 of calcification with 50 µM Fe in the presence of high Pi-challenge resulted in a significant induction of de novo synthesis of the two proteins GAS6 and AXL that reached levels not statistically different from control samples (+6.90% GAS6; +12.66% AXL; Pi + Fe compared to Pi day 9; *p* < 0.05; Figure 6).

### 2.3. Ferric Citrate Induces Autophagy in Established Calcified VSMCs

**Figure 7:**
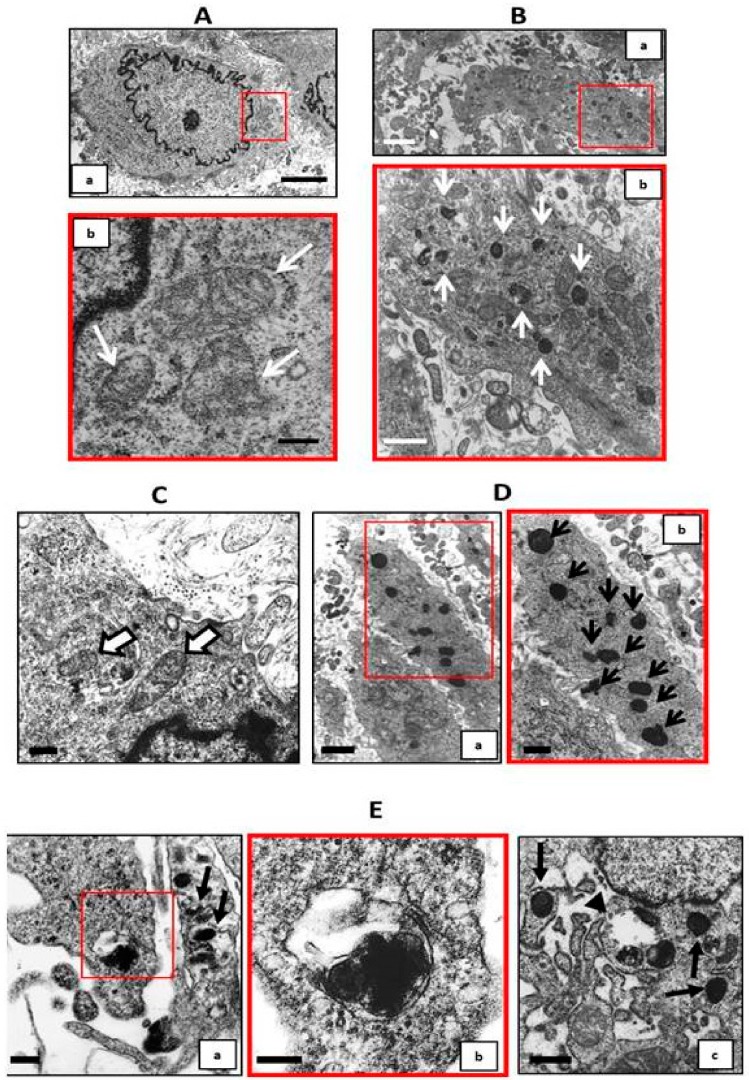
Effect of the therapeutic addition of ferric citrate on autophagosome formation during the progression of high Pi-induced calcification. (**A**) Image a: Electron microscopy micrograph of an ultra-thin section from control VSMCs on day 9; scale bar 2 µm. Image b: Enlargement of red boxed area in image a showing normal mitochondria (arrows); scale bar 200 nm. (**B**) Image a: Electron microscopy micrograph of an ultra-thin section from 5 mM Pi treated VSMCs for 9 days; scale bar 1 µm. Image b: Enlargement of red boxed area in image a, arrows point to calcified mitochondria; scale bar 500 nm. (**C**) Electron microscopy micrograph of an ultra-thin section from control VSMCs on day 14 showing normal mitochondria (arrows); scale bar 200 nm. (**D**) Image a: Electron microscopy micrograph of an ultra-thin section from 5 mM Pi treated VSMCs for 14 days; scale bar 1 µm. Image b: Enlargement of red boxed area in image a, arrows point to completely calcified mitochondria; scale bar 500 nm. (**E**) Image a: Ultra-thin section from 5 mM Pi + 50 µM Fe treated VSMCs from day 9 to day 14, arrows point to autophagic vacuoles; scale bar 200 nm. Image b: Enlargement of the red boxed area in image a shows an autophagic vacuole with completely calcified mitochondria inside; scale bar 100 nm. Image c: Arrowhead points to an autophagic vacuole with completely calcified mitochondria inside, whereas arrows point to calcified mitochondria; scale bar 400 nm.

Using electron microscopy, we analyzed the VSMC finding that in calcified conditions, there were several partially- or completely-calcified mitochondria that lost the cristae structure which is typical of functionally-intact mitochondria (Figure 7A–C; Figure 7B,D inset b). It has been demonstrated that high Pi-induced calcification inhibits the autophagic process, and in fact, in 9 and 14 days’ calcified VSMCs, a few or no autophagosomes were detectable within the cells, despite the many calcified mitochondria (Figure 7B,D). Treating calcified VSMCs with 50 µM Fe from day 9 to day 14 of calcification induced autophagosomes formation (Figure 7E inset b). In fact, together with the calcified mitochondria free in the cytoplasm, it was possible to detect some of them rounded by a double layer membrane, which is typical of autophagosome (Figure 7E inset b).

**Figure 8:**
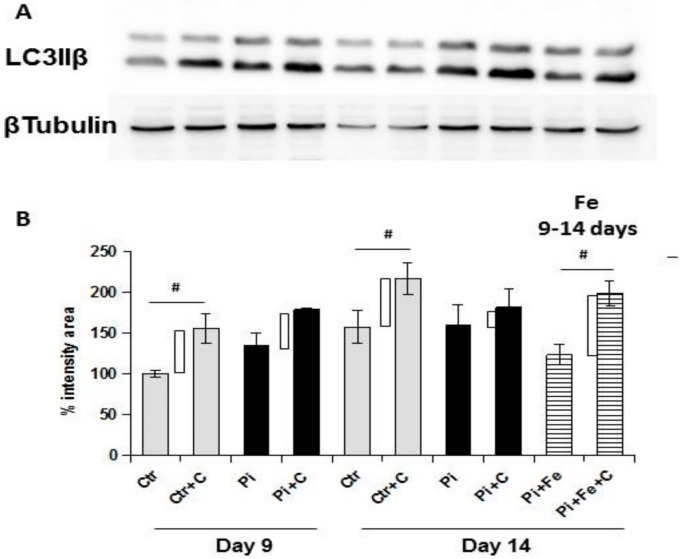
Effect of therapeutic addition of ferric citrate on autophagic flux during the progression of high Pi-induced calcification. Rat VSMCs were challenged with 5 mM Pi for up to 14 days. (**A**) LC3IIB protein expression was detected by immunoblotting analysis in the absence or presence of ON treatment with 25 µM chloroquine ‘C’ from day 8 or 13. Lane 1: Control day 9; lane 2: Control day 9 + chloroquine; lane 3: 5 mM Pi day 9; lane 4: 5 mM Pi day 9 + chloroquine; lane 5: Control day 14; lane 6: Control day 14+chloroquine; lane 7: 5 mM Pi day 14; lane 8: 5 mM Pi day 14+chloroquine; lane 9: 5 mM Pi + 50 µM Fe from day 9 to 14; lane 10: 5 mM Pi + 50 µM Fe from day 9 to 14+chloroquine. Representative result of one of three different experiments. (**B**) Graph of the immunoblot band intensity is shown normalized for beta tubulin area intensity. Shaded bars represent different conditions in presence of ON treatment with chloroquine. Empty rectangles indicate the autophagic flux. Data are presented as the mean ±SE (# *p* < 0.05).

In order to confirm the electron microscopy observations, we studied the autophagic flux by the analysis of the marker of the autophagosome light-chain 3-IIB (LC3IIβ). We compared the LC3IIβ expression in samples with or without treatment with 25 µM chloroquine, an inhibitor of autophagosome degradation (Figure 8A). Analyzing the difference of LC3IIβ expression between the samples with and without chloroquine addition, we found that Pi challenge inhibited the autophagic flux progressively from day 9 to day 14 of calcification, as expected (43.4 and 20.8 Δ intensity area, Pi day 9 and 14, respectively; *p* < 0.05; Figure 8B). In contrast, treatment of calcified cells with 50 µM Fe from day 9 to day 14 of calcification induced an autophagic flux that was significantly increased compared to high Pi-treated samples on day 14 and comparable to controls (59.7, 20.8 and 75.6; Δ intensity area, ctr, Pi and Pi+Fe day 14, respectively; Pi + Fe vs Pi^a^ and Pi + Fe vs ctr^b^; ^a^ *p* < 0.05; ^b^ ns; Figure 8B). This result confirms the induction of autophagy in samples therapeutically treated with 50 µM Fe.

## 3. Discussion

VC is a multifactorial process due to the pathological deposition of minerals in the vascular system. Several causes have been identified in the pathogenesis of VC, such as an imbalance between pro- and anti- calcific factors; osteo-chondrogenic differentiation of VSMCs induced by inflammation, lipids, calcium and phosphate; apoptotic process; matrix degradation; and elastolysis [10].

In CKD, together with the traditional factors inducing VC, some nontraditional factors exist, namely uremic milieu and hyperphosphatemia. It is accepted that among all of the pathophysiologic mechanisms in CKD, VC is strongly associated with CV mortality, and it appears that high serum Pi levels are the most likely culprit behind VC [11].

Many efforts have been made in recent years to try to properly investigate and elucidate the mechanisms of high Pi-induced VC. The most in vitro-utilized approach is to study the effect of different molecules on the prevention of VC through different aspects such as calcium deposition, VSMCs osteoblastic transdifferentiation, apoptosis, elastolysis, matrix degradation, and authophagy. Applying the prophylactic approach in vitro, we published a study on the effect of iron on VC, demonstrating that ferric citrate prevents high Pi-induced calcium deposition by preventing apoptosis and by the potentiation of authophagy [8]. Interestingly, we also found that iron acts on the extracellular matrix osteo-chondrogenic shift induced by high Pi treatment, both preventing and blocking/partially reverting it [9]. The interest in the action of iron on calcification is also legitimated by the recent clinical use of this molecule as a Pi binder. In fact, two new Ca-free, iron-based P binders are now available to treat hyperphosphatemia in CKD, namely iron citrate and sucroferric oxyhydroxide [7].

Trying to investigate the direct effect of iron on VC, in the present study, we evaluated the effect of ferric citrate on high Pi-induced calcification mechanisms in established calcified VSMCs, elucidating the data of the effects of iron on calcium deposition [8]. We performed an in vitro therapeutic approach to evaluate how iron can modulate calcification in an established pathological model of VC. The addition of ferric citrate to 7-day calcified VSMCs completely blocked additional calcium deposition induced by high Pi in the following 8 days of treatment. This result is highly surprising, considering that after 7 days of high Pi challenge, the VSMCs that received iron were already transformed into simil-osteoblasts, and actively deposited calcium-phosphate crystals [8]. In contrast, in the prophylactic approach, when VSMCs receive high Pi and iron, their phenotype is muscular, and iron may act on the prevention of high Pi-induced VC mechanisms. Thus, performing experiments with the prophylactic and therapeutic administration of iron, we demonstrate that iron can completely block calcium deposition regardless of the grade of high Pi-induced simil-osteoblastic transformation of VSMCs.

Trying to elucidate the mechanisms responsible for this therapeutic action, we studied apoptosis and autophagy, since we already demonstrated their involvement in the prevention of high Pi-induced calcification [8]. Analyzing apoptosis in the early phase, we found that iron treatment for 4 days on high Pi-calcified VSMCs blocked the progression of apoptosis with a number of total apoptotic cells which was not statistically different from that at the beginning of the addition of iron. These data are surprising if we consider that iron is added together with high Pi, and that in 11-day, high Pi-treated VSMCs, apoptotic cell number increases. Thus, iron is able not only to prevent high Pi-induced apoptosis, but also to delay and block the progression of apoptosis, even if the process is already started and the apoptosis-inducing agent is present. Confirming the anti-apoptotic effect of iron are the data on H_2_O_2_, a recognized proapoptotic agent. After 7 days of high Pi treatment, H_2_O_2_ induced a worsening of calcification that was completely counteracted by iron. Interestingly, even a concentration of iron of 2 μM, i.e., one that is ineffective in preventing high Pi calcification, was able to completely prevent a H_2_O_2_-induced increase in calcium deposition. Moreover, iron is also effective at blocking H_2_O_2_-induced exacerbation of calcification on already calcified VSMCs, demonstrating an iron anti-apoptotic effect in our in vitro experimental model.

Study of the late stages of the apoptotic process by TUNEL confirms this concept. In fact, increased apoptotic nuclei were detected during high Pi treatment with a massive release of fragmented apoptotic bodies at late time points [12]. Even if in this work only nuclear bodies were taken into account in evaluating apoptosis, fragmented red dust, at the cytoplasmic level and in the extracellular matrix, was detected. This kind of signal can be interpreted as fragmented apoptotic bodies excreted from dying cells in the extracellular space and/or possibly taken up by phagocytic cells [13]. We decided to evaluate only the nuclear fragmentation of nuclei during apoptotic death, but interestingly, the specific increase of nuclear red signals consensually paralleled the increase of extra-nuclear apoptotic signals, thus confirming the pattern of apoptosis during high Pi and Fe treatment.

We next investigated the GAS6/AXL prosurvival pathway, which has been shown to be affected by high Pi treatment [13]. Our data confirmed the involvement of these proteins in the iron citrate protective mechanism. In fact, we found an iron-induced positive modulation of GAS6/AXL synthesis. In our previous study on prophylactic treatment with iron, we demonstrated a preventative action exerted by iron on high Pi-induced GAS6/AXL down-regulation. Interestingly, therapeutic-added iron not only protects by further high Pi-induced down-regulation of GAS6/AXL proteins, but strongly stimulates their de novo synthesis, reactivating this prosurvival and anti-apoptotic pathway. Therefore, one of the mechanisms by which iron citrate exerts its anti-apoptotic action could be the restoration of the GAS6/AXL prosurvival axis. Apoptosis is a relevant process to VC. In fact, apoptotic cells and released apoptotic bodies form a nidus for calcification, and increase the propensity of VSMCs to calcify after calcium and phosphate treatment [14]. Apoptosis, in calcifying VSMCs, is driven mainly by calcium rather than phosphate alone, as in our model. Nevertheless, a source of calcium is calcium phosphate crystals that undergo lysosomal degradation by VSMCs, leading to very high intracellular calcium levels and subsequent cell death, beginning a vicious cycle of progressive calcification [15].

In this study, we show that one of the mechanisms of high Pi-induced calcification, apoptosis, can be reverted and counteracted by iron added therapeutically in vitro. These findings demonstrate that iron has an anti-apoptotic effect in high Pi-stimulated VSMCs, independent of the grade of calcification and simil-osteoblastic cell transformation.

Apoptosis is strictly linked to autophagy, and can be considered as a dramatic consequence of the failure of autophagy to re-establish a positive balance for the cells evolving in survival. Since iron is prophylactically able to modulate autophagy in high Pi-treated VSMCs, we investigated the autophagic process. Autophagy is a highly-conserved cellular process which is responsible for the removal or recycling of long-lived proteins and organelles aimed to give an alternative source of nutrients to cells in conditions of either starvation or stress [16]. To some extent, autophagy can prevent the activation of apoptotic pathways through the removal of damaged mitochondria [17], although, in some systems, autophagy can enhance an apoptotic response [18]. It has been demonstrated that autophagy is a protective phenomenon with respect of high Pi-induced calcification, playing a pivotal role in arterial calcification [19]. We found that the therapeutic addition of iron was able to stimulate autophagy, even in the presence of high Pi challenge on already calcified VSMCs. In fact, from day 9 to day 14 of calcification, there was a significant reduction of autophagic flux, and treating cells in the same 6 days with iron, even in presence of high Pi, resulted not only in the prevention of further autophagic flux downregulation, but also in the re-induction of the autophagic process. These data were confirmed by electron microscopy. In fact, at 9 days of calcification, there were many calcified mitochondria and no or few autophagosomes, showing the degree of difficulty for the cells to remove these completely damaged organelles. After 6 days of therapeutic iron treatment, on day 14 of calcification, there were still many calcified mitochondria, but there were also autophagosomes filled with damaged organelles, indicating that iron reactivates the autophagic process, helping cells to eliminate damaged mitochondria. As demonstrated for apoptosis, also with respect to autophagy, the therapeutic addition of iron is able to revert the high Pi-induced decrease of the autophagic flux, reactivating the autophagic process.

Iron is an oxidative agent and is rapidly stored by cells because of its potential toxic effect. The addition of iron citrate may affect the red-ox state of high Pi-stimulated VSMCs, and a link between oxidative stress and autophagy in different cellular models has been demonstrated. In fact, autophagy is induced as a response to the toxic effect of reactive oxygen species (ROS), and contributes to clearing the cells of all irreversibly-oxidized biomolecules (proteins, DNA and lipids); this is why it can be included in antioxidant and DNA damage repair systems [20]. The balance between ROS production and cellular antioxidant mechanisms in high Pi-stimulated VSMCs has been poorly investigated. Probably, a better understanding of oxidative stress/antioxidant defense processes in VC is needed to determine whether iron’s oxidant nature is linked to its mechanism of action and to its effect on autophagy and apoptosis. An important observation is that iron entry into cells induces ferritin synthesis for the storage of this ion to prevent the Fenton reaction, which is dangerous for cell viability due to the production of free radicals. Interestingly, there is evidence that the feroxidase activity of the ferritin heavy chain can protect from both calcification [21,22] and apoptosis [23]. Thus, further studies are needed to deeply investigate whether ferritin heavy chain feroxidase activity can account for the block of calcium deposition and the anti-apoptotic and proautophagic effects of iron in our model of therapeutic in vitro progression of high Pi-induced calcification.

In summary, we studied the effect of iron treatment on established calcified VSMCs, and consequently, on the progression of calcification in vitro. We demonstrated that iron blocks the progression of calcium deposition by affecting apoptosis and autophagy. In fact, iron citrate can revert the high Pi-induced apoptotic process and re-induce autophagy; the combination of these two effects is probably responsible for blocking the calcification progression. These findings, together with the data on iron’s ability to partially revert the high Pi-induced osteo-chondrogenic shift of the VSMC extracellular matrix, help to better elucidate the complex effects of iron and add a piece to the mosaic of its action.

VSMCs are cells with some plasticity, and share their mesenchymal origin with osteoblasts. This may explain why continuous exposition at high Pi levels induces a VSMC simil-osteoblastic differentiation and their ability to deposit calcium phosphate crystals in the vasculature. Nevertheless, we have already demonstrated that suspending the high Pi challenge for brief periods is enough to significantly decrease calcium deposition, meaning that VSMCs can stop the calcium deposition activity, even if already transformed, if the pro calcifying stimulus is suspended, even temporally, at least in vitro [24]. Here we demonstrate that even if VSMCs are already transformed and the procalcifying stimulus is present, reverting apoptosis and inducing autophagy probably contribute to stopping calcium deposition. Taken together, these data suggest that already transformed VSMCs are prone to stopping the calcium deposition process if calcification mechanisms are adequately targeted.

From these in vitro data, we can conclude that iron can block the progression of calcification and, in line with the iron effect on the extracellular matrix osteochondrogenic shift, we demonstrate that some high Pi-induced processes can be reverted, not only prevented, in vitro. The importance of this reversion concept is in the arrest of additional calcium deposition that is fundamental if we consider the detrimental role of VC in CV disease and its association with increased mortality in CKD patients.

## 4. Materials and Methods

### 4.1. Materials

DMEM (high glucose, [4.5 g/L]), NaCl, FBS, and a BCA protein assay kit (Pierce, Rockford, IL, USA) were purchased from Euroclone (Milan, Italy); Na_3_PO_4_, MgSO_4_, NaH_2_PO_4_, KH_2_PO_4_, and KCl were purchased from Carlo Erba (Milan, Italy); ferric citrate, Trypsin-EDTA-Solution (T4174) and Collagenasi type IA (C9891) were purchased from Sigma (St. Louis, MO, USA); the primary antibody for Axl (sc-1097) and GAS6 (N-20 sc-1936) were purchased from Santa Cruz (Heidelberg, Germany); the primary antibody for LC3-II was purchased from Cell Signalling (Danvers, MA, USA), and the anti-rabbit secondary antibody was purchased from GeneTex (Irvine, CA, USA); the anti-goat secondary antibody (ab6741) was purchased from Abcam (Cambrige, UK); Hepes Buffer Solution, PVDF membrane Invitrogen/Applied Biosystem (Milan, Italy); PE Annexin V Apoptosis Detection Kit I (559763) was purchased from Bioscience. An ApopTag Red In Situ Apoptosis Detection kit S7165 was purchased from Chemicon. All other reagents were obtained from Sigma (St. Louis, MO, USA).

### 4.2. Methods

#### 4.2.1. Induction of Calcification

Rat VSMCs were obtained by enzymatic digestion, as previously described [25], and were routinely subcultured in a growth medium (DMEM containing 10% FBS supplemented with 100 U/mL penicillin, 0.1 mg/mL streptomycin). At 80% confluence, cells were switched to the calcification medium (DMEM containing 12% FBS supplemented with 100 U/mL penicillin, 0.1 mg/mL streptomycin, 10 mM sodium pyruvate, and 50 μg/mL ascorbic acid) and challenged with 5mM Na_3_PO_4_ (Pi) for up to 15 days. Ferric citrate (Fe) and H_2_O_2_ were added therapeutically on different days (7 or 9) after Pi challenge, and samples treated with Fe or H_2_O_2_ or both, always in presence of Pi, were then processed at the final time point required (day 11, 14, or 15). In H_2_O_2_ experiments on calcification, cells were stimulated with 4.5 mM Pi. The medium was replaced every 2 or 3 days. Cells were used between the sixth and eight passage.

To analyze the ex vivo calcification of rat aortic rings, we harvested aorta from female Lewis rats (120-150 g). Briefly, after scraping adipose tissue from the adventitia, the cleaned aorta was cut into 3 mm-thick sections and stabilized in a growth medium for 24 h in petri dishes previously coated with 100 µg/mL fibronectin. After stabilization, the medium was replaced with a calcification medium (3 mM Pi) which was changed every 2 or 3 days up to day 14. Male Sprague-Dawley rats were kept in accordance with guidelines from Directive 2010/63/EU of the European Parliament on the protection of animals used for scientific purposes and the Italian Ministry of Health and the local University of Milan ethics committee approved the protocol (NCT03169400; date of approval: 30 May 2017)

#### 4.2.2. Quantification of Calcium Deposition and Alizarin Red Staining

The quantification of calcium deposition was performed by Alizarin Red S staining, followed by HClO_4_ destaining. Extracellular calcium deposits were stained with an Alizarin Red S solution for 30 minutes and destained for 24 h with 5% perchloric acid. The Ca content was determined calorimetrically at a wavelength of 450nm. Protein content was quantified using a BCA protein assay kit, and Ca deposition was normalized to protein content and expressed as absorbance (OD/mg protein).

To visualize calcium deposition, cells were grown on plastic supports and, at the end of the experiment, were fixed with 70% EtOH and stained with 1 mg/mL Alizarin Red S solution for 30 minutes. Cells were rinsed and Ca deposition was photographed. The deposition of calcium salts by Alizarin Red S staining was evaluated as the presence of fine granules (score 1) or large calcific deposits (score 2), or a confluent structure of calcium deposits (score 3); 0 was referred to as the absence of calcium deposits; a.s. arbitrary score.

#### 4.2.3. Western Blot

Rat VSMCs were harvested in an ice-cold homogenization buffer (50mM Tris pH 8 with 0.5% IGEPAL CA-630, 1 mM phenylmethanesulfonyl fluoride (PMSF), 1mM benzamidine HCl, 1mM sodium fluoride (NaF), 10mM β-glycerophosphate, and complete protease inhibitor), freeze-thawed 2X and sonicated 5 × 20 s at 40% power. Samples were then centrifuged at 13,000 g for 15 minutes at 4 °C, and the protein concentration was measured. Denatured samples (50 μg of total protein) were separated by electrophoresis on a 12% (LC3-II), 7.5% (Axl and GAS6) SDS-polyacrylamide gel, and were then transferred to the PVDF membrane. The membranes were incubated 1 h with 1:200 (Axl) or O/N with 1:100 (GAS6), 1:300 (LC3-II) primary antibodies, followed by 1 h incubation 1:10,000 (Axl and GAS6) peroxidase-conjugated anti-goat or 1:20,000 (LC3-II) peroxidase-conjugated anti-rabbit secondary antibodies. Protein bands were visualized using an ECL detection kit, and area intensity was measured.

#### 4.2.4. Detection of Apoptosis

To evaluate apoptosis by cytofluorimetry with a PE Annexin V Apoptosis Detection Kit, we disaggregated the VSMC multilayer. After two washes with warm PBS pH 7.4, we incubated the sample with 1 ml of warm collagenase solution (5 mg/mL with 2.7 mM CaCl_2_) for 4 min at 37 °C. Then, we added 1 ml of warm trypsin-EDTA solution (1×) for 1 min at 37 °C, and cells were gently disaggregated mechanically. Successively, we washed and resuspended the cells at 750,000/sample to be processed as indicated by the PE Annexin V Apoptosis Detection Kit I protocol. Briefly, VSCM cells (750.000/sample) were resuspended in 1× Binding Buffer, 100 µL, and stained with 10 µL PE annexin V and 10 µL 7-AAD for 30 min at room temperature in the dark. Following the addition of 0.4 mL of 1× Binding Buffer, fluorescence of annexin V and 7-AAD were detected in the FL-2 and FL-3 channels, respectively, within 1 h using a FACS Verse (BD Biosciences, Rockville, MD, USA). Unstained and untreated cells were used to establish the instrument settings.

Apoptosis was also evaluated histologically by DNA fragmentation with the TUNEL technique, both in cells and in aortic rings, with the ApopTag Red in situ Apoptosis Detection Kit, following the manufacturer’s instructions. Apoptosis was detected as red signals mainly sited in the nuclei of VSMCs. A few apoptotic bodies were also observed in the extracellular matrix and in the cytoplasm, but only nuclear signals were considered in the present work. The number of apoptotic nuclei in 2 HPF (high power field, X40) was recorded for each experimental condition and scored as follows: 1: <5% apoptotic nuclei, 2: <15% apoptotic nuclei; 3: <25% apoptotic nuclei; 4: ≥25% apoptotic nuclei.

#### 4.2.5. Electron Microscopy

Samples of cells were scraped from culture flask, fixed in 2.5% glutaraldehyde in 0.13 M phosphate buffer at pH 7.2–7.4 for 2 h, post-fixed in 1% osmium tetroxide, dehydrated through graded ethanol and propylen oxide, and embedded in an epoxy resin. Ultrathin sections (50–60 nm thick) were counterstained with uranyl acetate and lead citrate for observation with a Jeol JEM 1010 transmission electron microscope (Jeol, Tokyo, Japan).

#### 4.2.6. Statistical Analysis

The results were expressed as mean ± SEM. Each experiment was performed at least three times, at least in triplicate. The differences between groups were analyzed by one-way ANOVA followed by a Bonferroni post-hoc test, and were considered statistically significant when *p* value < 0.05. A score statistical analysis was evaluated by a Kruskal-Wallis test, corrected for ties.
